# The Antioxidant Action of *Astragali radix*: Its Active Components and Molecular Basis

**DOI:** 10.3390/molecules29081691

**Published:** 2024-04-09

**Authors:** Juan Yao, Ting Peng, Changxin Shao, Yuanyuan Liu, Huanhuan Lin, Yongqi Liu

**Affiliations:** 1 College of Pharmacy, Gansu University of Chinese Medicine, Lanzhou 730101, China; pengting0930@foxmail.com (T.P.); scx129@126.com (C.S.); 18893705893@163.com (H.L.); 2 College of Basic Medicine, Gansu University of Chinese Medicine, Lanzhou 730013, China; 17355137976@163.com

**Keywords:** *Astragali radix*, antioxidant, astragalus polysaccharides, flavonoids compounds, saponins compounds

## Abstract

*Astragali radix* is a traditional medicinal herb with a long history and wide application. It is frequently used in prescriptions with other medicinal materials to replenish Qi. According to the classics of traditional Chinese medicine, *Astragali radix* is attributed with properties such as Qi replenishing and surface solidifying, sore healing and muscle generating, and inducing diuresis to reduce edema. Modern pharmacological studies have demonstrated that some extracts and active ingredients in *Astragali radix* function as antioxidants. The polysaccharides, saponins, and flavonoids in *Astragali radix* offer beneficial effects in preventing and controlling diseases caused by oxidative stress. However, there is still a lack of comprehensive research on the effective components and molecular mechanisms through which *Astragali radix* exerts antioxidant activity. In this paper, we review the active components with antioxidant effects in *Astragali radix*; summarize the content, bioavailability, and antioxidant mechanisms; and offer a reference for the clinical application of *Astragalus* and the future development of novel antioxidants.

## 1. Introduction

*Astragali radix* is the dried root of the leguminous plant *Astragalus membranaceus* (Fisch.) Bge. var. *Mongolicus* (Bge.) Hisao or *Astragalus membranaceus* (Fisch.) Bge., widely distributed throughout the temperate regions of the world. As a typical traditional Chinese medicine (TCM), with medicine and food homology, *Astragalus radix* is widely used in the clinical treatment of TCM, food, health products, cosmetics, and animal husbandry [1,2]. For example, *Astragalus* extracts added to cosmetics have the effect of anti-aging. Moreover, adding *Astragalus* or its extracts to animal feed can improve the immunity and growth performance of grass carp, tilapia, and broiler, increase meat yield, and reduce the use of antibiotics in the feeding process [3,4]. Modern pharmacological studies have revealed that *Astragalus* and its extracts have pharmacological effects such as being antioxidant, hepatoprotective, nephroprotective [5], anti-aging, anti-tumor, and antiviral, as well as contributing to immune regulation and cardiovascular protection [6]. Owing to its outstanding clinical efficacy and low side effects, *Astragali radix* has been widely utilized. In this review, the authors examine the extraction and structural composition of *Astragalus* as well as its effects on antioxidants. Furthermore, the latest research and progress on the pharmacological effects of *Astragali radix* are illustrated to provide a theoretical basis for the oxidative-stress-induced disease application of *Astragali radix*.

## 2. Components and Contents of *Astragali radix* with Antioxidant Activity

*Astragalus* is composed of a variety of chemical components, including *Astragalus* polysaccharide (APS), saponins (such as Astragaloside I–IV), and flavonoids (Formononetin, Isorhamnetin, Quercetin, Kaempferol, Calycosin, etc.) [7,8,9]. Additionally, *Astragalus* contains various amino acids (such as asparagine, aspartic acid, proline, arginine, and alanine), trace elements (including Cr, Mn, Cu, Fe, Zn, Se, and Cs), folic acid, coumarin, and betaine. A recent study found that *Astragalus* contains an alcohol-soluble polysaccharide (ASP), which is a neutral polysaccharide composed of glucose, arabinose, mannose, and galactose. The study also revealed that the total sugar, uronic acid, and protein contents of ASP were 92.04%, 1.42%, and 0.51%, respectively [10]. Another study showed that the APS content is closely linked to the place of origin and growth year. Among the four major *Astragalus*-producing areas in China (Heilongjiang, Inner Mongolia, Shanxi, and Gansu), Gansu exhibited the highest APS content of *Astragalus*. The study also found that the APS content of one-year-old *Astragalus* was the highest, decreasing with the extension of growth years [11]. Samuel et al. [12] utilized an automatic amino acid analyzer and AlCl_3_ colorimetric method to detect the contents of total amino acids, total flavonoids, and polysaccharides in the dried roots of *Astragalus*. The result showed that the total flavonoid, polysaccharide, and amino acid contents were 1.90%, 20.44%, and 8.89%, respectively. Yao et al. [13] used HPLC-CAD to quantitatively detect five compounds in *Astragalus*, including Formononetin (FMN), Calycosin-7-O-β-D-glucopyranoside (CG), Astragaloside I (AS-I), Astragaloside II (AS-II), and Astragaloside IV (AS-IV). The content of AS-I was 0.306~0.922 mg/g, while the contents of AS-II, AS-IV, CG, and FMN ranged from 0.053 to 0.092 mg/g, 0.015 to 0.092 mg/g, 0.0069 to 0.823 mg/g, and 0 to 0.098 mg/g, respectively. Research showed that some components of *Astragalus membranaceus* exhibit excellent antioxidant effects, in particular, on experimental models.

## 3. Bioavailability of *Astragali radix* Extracts and Its Constituents

Bioavailability studies of *Astragali radix* and its constituents have mainly been performed on animals. After the oral administration of 20 mg/kg of AS-IV (dissolved in ethanol and diluted in normal saline), the highest plasma concentration of AS-IV was 0.38 μg/mL in the intestinal perfusion model of rats. In addition, the absolute bioavailability of AS-IV (2.5 mg/kg) was 2.2% in the intestinal perfusion model of rats after tail vein injections [14]. Beagle dogs [15] were administered a single oral dose of 10 mg/kg or three intravenous doses of AS-IV at 0.5, 1, and 2 mg/kg. The peak concentration was observed 1 h after intravenous administration, with a maximum blood concentration of 1.624 μg/mL. The absolute bioavailability after oral administration was 7.4%.

In a research study [16], rats were given FMN through a 50 mg/kg oral administration dose or tail vein injection at 10 mg/kg. The results showed that the unchanged/free FMN oral bioavailability was quite low (~3%). This could be due to the comprehensive first-pass metabolism of phase I oxidative metabolism and phase II glucuronidation and/or sulfation in the liver and intestine. In a separate study [17], researchers administered 20 mg/kg of FMN or ononin orally to rats and found that the FMN absolute bioavailability was 21.8%, while the bioavailability of ononin was much lower at 7.3%. In a study conducted by Tao et al. [18], rats were administered an *Astragalus* aqueous extract (the contents of AS-I, AS-II, AS-IV, CG, ononin, and FMN were 41.45, 152.90, 69.42, 52.76, 29.87, and 15.07 μg/mL, respectively) via gavage to investigate the pharmacokinetics of CG, ononin, FMN, AS-IV, AS II, and AS-I. The findings revealed that all six components reached their maximum blood concentrations within 1 h, with values ranging from 727.08 ± 61.34 ng/mL to 3566.08 ± 401.91 ng/mL. Specifically, the highest blood concentrations were observed for AS-II at 3566.08 ± 401.91 ng/mL, followed by CG at 1526.43 ± 290.99 ng/mL, AS-IV at 1442.51 ± 166.16 ng/mL, ononin at 889.29 ± 135.89 ng/mL, AS-I at 727.08 ± 61.34 ng/mL, and FMN at 1190.35 ± 199.83 ng/mL. In a separate study [19], rats were administered a single dose of CG either intravenously (0.5 mg/kg) or orally (10 mg/kg). The results showed that oral administration resulted in a significantly higher Cmax (1189.66 ± 346.95 ng/mL) compared to intravenous administration (965.24 ± 133.53 ng/mL). However, the absolute oral bioavailability was found to be only 0.304%.

Song et al. [20] used an in vitro simulated digestion model to explore the transformation process of *Astragali radix* extract in the gastrointestinal tract after oral administration. It was found that the saponins were transformed in the oral, S-intestinal, and L-intestinal stages. A variety of Astragaloside glycosides are eventually converted to AS-IV, and flavonoid glycosides are converted to aglycones through a complex and regular series of deglycosylation. Flavone is mainly degraded in the L-intestine stage. Research conducted on tissue distribution revealed that AS-IV was present in various tissues of mice following intravenous tail administration. The lung and kidney were identified as the primary distribution organs that are potentially linked to the transportation of the circulatory system and *Astragalus* into the lung meridian [21]. AS-IV was found to be present in the brain of mice, indicating its ability to cross the blood–brain barrier [21]. In recent years, it has been found that Astragalosidic acid (LS-102) is a new water-soluble derivative of AS-IV. Sun et al. [22] administered three doses (5.0, 10.0, and 20.0 mg/kg) of LS-102 orally to beagle dogs and conducted pharmacokinetic studies. The results showed that the three doses of LS-102 reached the peak plasma concentration about 2 h after oral administration, with a half-life ranging from 1.55 to 4.49 h. In another study [23], after oral administration of 20 mg/kg of LS-102 and AS-IV, it was found that LS-102 was rapidly absorbed, reaching a maximum concentration of 248.7 ± 22.0 ng/mL 1.0 ± 0.5 h after oral administration. The relative bioavailability of LS-102 is twice that of AS-IV. Calycosin (CA) is predominantly distributed in the kidney, heart, and liver tissues of rats. Its main metabolic pathways involve glucuronidation and sulfation, with Ca-3-sulfate as the main metabolite [24]. In one study [25], two groups of rats were given 0.1 or 1% Quercetin (Que) in their diet for 11 weeks. It was found that Que and its main metabolites, Isorhamnetin (ISO) and Tamarix, had the highest concentrations in the lungs of rats. The liver and kidney, which are the main metabolic organs of Que, contained moderate concentrations of Que, ISO, and Tamarix. In addition, APS, another active component in *Astragalus*, also exhibited low bioavailability due to its high molecular weight, low solubility, and negative charge [26].

## 4. Biology of *Astragali radix* Extract and its Components: The Main Mechanism of Antioxidant Action

**Figure 1:**
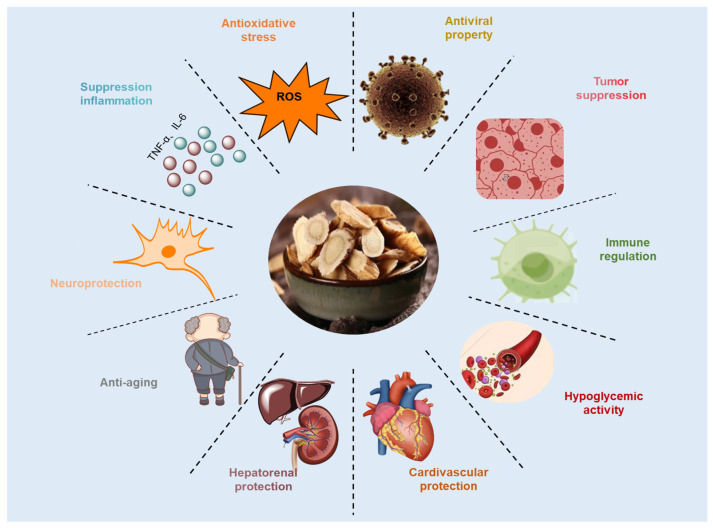
Main biological effects of *Astragali radix* extracts and their bioactive compounds.

*Astragali radix* extract and its bioactive components have been found to have a wide range of beneficial effects under both normal and pathological conditions (Figure 1). These bioactive ingredients are excellent antioxidants, immunomodulators, and tumor inhibitors [5]. Additionally, they have significant effects on liver and kidney protection [5] and anti-aging, antiviral, cardiovascular protection [6], neuroprotection [27], hypoglycemia, and anti-inflammatory [28] effects. *Astragalus* contains active ingredients that could help lower blood pressure and reduce levels of triglyceride and total cholesterol in rats [29,30]. Studies have shown that these effects may also have therapeutic benefits for hypertension and hyperlipidemia.

The bioactive compounds found in *Astragalus* possess several antioxidant mechanisms. These include reducing MDA/T-AOC levels, down-regulating the transcription of NOX2/4 mRNA and the expression of the NOX2 protein [31], promoting the activation of Nrf2 and the expression of downstream antioxidant factors [32], and enhancing the expression of various antioxidant enzymes in the body to achieve antioxidant effects. In vitro experiments have demonstrated that *Astragalus* exhibits excellent antioxidant activity. It has a strong scavenging capacity for DPPH and oxygen radicals [33]. Furthermore, some of the active ingredients in *Astragalus* can inhibit lipid peroxidation, chelate metal ions, and regulate oxidative-stress-related signaling pathways, thereby playing an antioxidant role. Xia et al. [34] treated a PD mouse model with an intraperitoneal injection of 100 mg/kg of AS-IV and found that AS-IV could reduce the production of reactive oxygen species (ROS) and the accumulation of damaged mitochondria, thereby alleviating the loss of dopamine neurons and deficits in the PD mouse model. Zhang et al. [35] administered oral doses of 20 and 40 mg/kg of Liquiritigenin (LQ) to mice with liver damage induced by arsenic trioxide (ATO) and found that LQ could inhibit ROS production and alleviate ATO-induced liver damage. The mechanism of action of LQ involves up-regulating the levels of antioxidant enzymes such as superoxide dismutase (SOD), catalase (CAT), and GSH. It also reduces the levels of inflammatory factors TNF-α and IL-6. LQ enhances the PI3K/AKT/mTOR signaling pathway, which, in turn, enhances autophagy. This mechanism helps alleviate the liver injury induced by ATO in mice. In one study [36], after treatment with 15 µg/mL of Calycosin-7-O-β-D-glucoside (CG), the cell viability of the oxygen–glucose deprivation/reperfusion (OGD/R) H22 model was significantly improved. Oxidative stress and cell apoptosis were significantly alleviated. It also up-regulated the expression of SIRT1, FOXO1, PGC-1α, and Bcl-2 and down-regulated the expression of Bax. This suggests that CG alleviates OGD/R-induced cell damage by inhibiting oxidative stress and activating the SIRT1/FOXO1/PGC-1α signaling pathway. In another study, Pan et al. [37] treated PC12 cells with 20 µM of CA and found that the formation of long amyloid fibrils was inhibited, and the amyloid species shifted to amorphous and non-toxic aggregates, which alleviated the neurotoxicity caused by α-synuclein amyloid fibrils. Moreover, CA recovered the activity of the GSH and SOD/CAT content and reduced the ROS content. These results suggest that CA may play an antioxidant role against the neurotoxicity induced by α-syn amyloids by restoring SOD/CAT activity and GSH content, reducing ROS content and caspase-3 activity, and changing the aggregation pathway to form non-toxic spices. Chen et al. [38] found that AD rats supplemented orally with 30 mg/kg of ononin could alleviate cognitive impairments and increase SOD and TAC levels in the brain tissue of AD rats. This suggests that ononin therapy can improve cognitive impairments and oxidative stress in AD rats. Sugimoto et al. [39] treated human neuroblastoma SH-SY5Y cells induced by hydrogen peroxide (H_2_O_2_) with 50 µM of FMN and found that FMN could alleviate cell death and reduce the increase in intracellular ROS levels induced by H_2_O_2_. Meanwhile, FMN enhanced the expression of antioxidant genes by activating PI3K/Akt-Nrf2 signaling. Sul et al. [40] pretreated LPS-induced lung epithelial cells with Que at different concentrations (0, 10, 20, 50, and 100 µM) and found that Que reduced intracellular ROS levels. Meanwhile, it inhibited the protein expression of NOX2 induced by LPS. This suggests that Que could reduce ROS-induced oxidative stress by inhibiting NOX2 production. In one study, Tie et al. [41] treated HepG2 cells induced by oleic acid (OA) with KAE at different concentrations (5, 10, and 20 µM) and found that KAE could reduce OA-induced lipid accumulation and oxidative stress in HepG2 cells. In another study, Alqudah et al. [42] administered 10 mg/kg of ISO to a high-fat diet and a streptozotocin-(HFD/STZ)-induced mice model of type 2 diabetes. It was found that ISO could increase GSH levels and decrease MDA levels, relieving the oxidative stress of diabetic mice. Shi et al. [43] treated H_2_O_2_-induced PC12 cells with different concentrations (2, 4, and 8 µM) of isoliquiritigenin and found that it could improve the activities of superoxide dismutase, glutathione peroxidase, and CAT. In addition, it could inhibit the release of lactate dehydrogenase and the production of ROS in cells to play a role in cell protection. Li et al. [44] pre-treated BALB/c and C57BL/6J mice with 100 mg/kg of AS-IV before exposing them to bright light and the DNA alkylating agent methyl mesylate (MMS). It was found that AS-IV could significantly alleviate the oxidative stress and DNA damage of mouse retina caused by bright light irradiation and MMS, thus preventing the degeneration of photoreceptors. Table 1 shows the structure of the main saponins and flavonoids of *Astragalus* and its antioxidant effects.

## 5. Antioxidative Mechanism of *Astragali radix*

Oxidative stress is a condition where there is an imbalance caused by the excessive production or delayed removal of toxic ROS, such as hydroxyl free radicals, superoxide, and hydrogen peroxide in the body [79]. Prolonged exposure to excessive ROS can cause damage to cellular macromolecules, including DNA, lipids, and proteins, ultimately leading to cell necrosis and apoptosis [80]. Mitochondria are the main places where organisms produce and supply energy. However, during this process, ROS are produced as a by-product of mitochondrial metabolism. As a result, mitochondria are the primary source of ROS within cells [81]. Enzymatic and non-enzymatic antioxidants are vital in protecting the body from oxidative stress. The enzyme antioxidant system includes several components, such as CAT, glutathione reductase (GR), glutathione peroxidase (GSH-Px), and SOD. On the other hand, the non-enzymatic endogenous antioxidant system mainly includes glutathione, vitamin C, and uric acid [82].

Like many other antioxidants derived from plants, the active compounds found in *Astragali radix* can scavenge free radicals, activate antioxidant defense systems, inhibit the activity of pro-oxidases, inhibit lipid peroxidation, chelate metal ions, and regulate signaling pathways.

### 5.1. Direct Scavenging Free Radicals In Vitro

Free radicals are produced by both endogenous and exogenous pathways. Endogenous free radicals are the products of aerobic cell metabolism, and exogenous free radicals are produced in a wide range of ways, which are mainly related to the external environment. The increased content of free radicals in the body plays a critical role in developing oxidative stress and various diseases. 

In their study, Samuel et al. [12] found that both water and alcohol extracts of *Astragalus* were effective at scavenging free radicals, with VC used as a positive control. The concentrations of bioactive components in the water extract and ethanol extract of *Astragali radix* were 38.26% and 32.11%, respectively. The antioxidant activities of the extracts were detected in vitro by ABTS, DPPH, and FRAP assays. The experimental results indicated that the alcohol extract of the *Astragalus* root exhibited an ABTS inhibition rate of 51.89% at a concentration of 20,000 μg/mL. On the other hand, the water extract demonstrated an inhibition rate of 56.52% in ABTS. When the concentration reached 1000 μg/mL, the ethanol and water extracts displayed inhibition rates of 70.94% and 69.24%, respectively, in DPPH. At a concentration of 20.0 μg/mL, the FARAP values of the ethanol and water extracts were 86.44 μg/mL and 51.33 μg/mL, respectively. These results indicate that *Astragalus* has excellent antioxidant activity and a certain scavenging effect on ABTS and DPPH free radicals. In their study, Wu et al. [5] investigated the antioxidant activity of the *Astragalus* extract through scavenging experiments on ABTS, DPPH, and hydroxyl radicals. They found that as the concentration of the *Astragalus* extract increased from 0.625 mg/mL to 10.000 mg/mL, the scavenging rates of DPPH and hydroxyl radicals reached 70.25% and 40.494%, respectively. However, the scavenging rates increased more slowly as the mass concentration increased further. As the concentration of the *Astragalus* extract increased from 0.625 mg/mL to 20.000 mg/mL, the scavenging rate of the ABTS free radical reached 70.25%. Moreover, the scavenging rate showed a slow increase with a further increase in the mass concentration.

Sheng et al. [33] utilized methanol to extract and isolate flavonoids from four different types of *Astragali radix* sourced from Shanxi, Inner Mongolia, Heilongjiang, and Gansu. The phenol contents were expressed as the milligram equivalent of gallic acid per 100 g of dry weight of *Astragalus* (mg GAE/100 g DW). The total flavone contents were expressed as milligrams of catechin equivalent per 100 g of dry weight of *Astragalus* (mg CE/100 g DW). The total phenolic content of the *Astragalus* root from four producing areas varied between 135.23 and 197.40 mg GAE/g, and Inner Mongolia had the highest content (197.40 ± 1.95 mg GAE/g). The content of total flavonoids of *Astragalus* from four producing areas was significantly different, ranging from 52.27 to 112.75 mg CE/g. Among them, the “Inner Mongolia” sample (112.75 ± 0.77 mg CE/g) was significantly higher than the other three producing areas. The researchers then evaluated the in vitro antioxidant capacity of these extracts using ORAC and DPPH radical scavenging assays. The results showed that the ORAC value of the *Astragalus* extract from Mongolia was the highest (628.94 ± 3.30 μmol TE/g), while the extract from Heilongjiang had the lowest value (471.29 ± 8.61 μmol TE/g). In the DPPH scavenging experiment, the extract of *Astragalus* from Heilongjiang exhibited the highest scavenging activity, with an IC_50_ value of 8.10 ± 0.54 μmol TE/g, while the extract from Gansu had the lowest IC_50_ value of 5.89 ± 0.36 μmol TE/g.

It can be observed that *Astragalus* and its various active ingredients exhibit a commendable ability to eliminate free radicals. Moreover, the antioxidant capacity varies across different producing locations and extraction methods, which could be attributed to the different content and physical and chemical properties of the antioxidant components present in *Astragalus*.

### 5.2. Improving the Activity of Antioxidant Enzymes

The body’s resistance to oxidative stress is heavily reliant on the endogenous antioxidant enzyme system, which consists of SOD, glutathione reductase, GSH-Px, CAT, and so on. SOD facilitates the spontaneous disproportionation of superoxide free radicals, resulting in the generation of H_2_O_2_. CAT and GSH-Px then work to eliminate H_2_O_2_ and prevent the cell damage caused by an excessive H_2_O_2_ concentration [83].

In a study conducted by Liu et al. [84], mice were pretreated with varying doses of APS (50 mg/kg, 100 mg/kg, and 200 mg/kg) before radiation exposure. The results showed that the APS groups had increased CAT, SOD, and GSH activities in their liver tissue and experienced less severe liver damage compared to the radiation group. This indicates that APS has a certain protective effect on liver oxidative damage caused by electromagnetic radiation. This protective effect is achieved by enhancing antioxidant enzyme activities, specifically SOD, GSH, and CAT. In a study by Zhou et al. [85], mice with alcoholic liver disease were given different doses of APS (300 mg/kg bw and 600 mg/kg bw) and *Astragalus* saponins (50 mg/kg bw and 100 mg/kg bw). It turned out that treatment with APS and *Astragalus* saponin could improve alcohol-induced liver injury and increase the activities of SOD, GSH, CAT, and GSH-Px in the liver of mice. 

In a tumor-bearing mouse model, APS was administered at concentrations of 20, 40, and 60 mg/mL in a volume of 100 mL via an intraperitoneal injection for 28 days. The treatment led to a decrease in the levels of myeloperoxidase (MPO), MDA, and NO and also increased the total antioxidant capacity and CAT, SOD, and GSH-Px activities in the mice with tumors [86]. The research found that increasing antioxidant enzyme activities such as SOD, CAT, and GSH-Px helped mitigate the oxidative stress caused by tumor-bearing states. A research study [87] found that, in rats, the total flavonoids of *Astragalus* flavones had a protective effect on cerebral ischemia-reperfusion injuries by improving the activity of GSH-Px and SOD.

### 5.3. Inhibiting Oxidase Activities

Peroxisomes are organelles in eukaryotic cells that contain one or more oxidase enzymes. These enzymes are involved in polyamine oxidation, fatty acid β oxidation, and the synthesis of plasma and bile acids and play a critical role in maintaining the redox homeostasis of organisms [88]. However, the enzymatic reaction catalyzed by oxidase is also a significant source of intracellular ROS, which have been found to play a significant role in the occurrence and progression of various diseases. Various enzymes, such as cytochrome P450, xanthine oxidase, NADPH oxidase, nitric oxide synthase, and mitochondrial oxidase, are commonly found in biological systems. Recent research suggests that *Astragalus* and its active components can act as antioxidants by inhibiting oxidase activity.

Xanthine oxidase (XOD) is a crucial enzyme that plays a vital role in the process of uric acid formation. By suppressing the activity of XOD, the production of uric acid can be reduced, which has a therapeutic effect on hyperuricemia [89]. Li et al. [90] conducted a study where they added APS into xanthine oxidase and measured the activity of XOD using ultraviolet spectrophotometry. Their findings revealed that APS had a certain inhibitory effect on XOD, with a dose-dependent manner in the concentration range of 0–0.4 mg/mL.

In both in vivo and in vitro settings, AS-IV has been found to inhibit the expression of NOX2 and NADPH oxidase 4 (NOX4). This leads to a reduction in the production of ROS, which ultimately helps to alleviate doxorubicin-induced myocardial injuries [91]. Additionally, in a study conducted on rats with normal blood pressure and spontaneously hypertensive rats, it was found that Que and its metabolites, ISO and Kaempferol (KAE), have the ability to inhibit NADPH oxidase activity in vascular smooth muscle cells. This inhibition occurs in a concentration-dependent manner [92]. In a separate study, Que was used to conduct in vitro experiments on major subtypes of the cytochrome P450 enzyme (CYP2C19, CYP2D6, CYP3A4, CYP2E1, and CYP2C9). The study revealed that Que had inhibitory effects on several subtypes of the cytochrome P450 enzyme and that these effects were dose-dependent within the concentration range of 1–150 μmol/L. Notably, CYP2C9 and CYP3A4 exhibited a particularly high sensitivity to Que, with IC_50_ values of 23.09 and 13.14 μmol/L, respectively [93]. In another study, Que was found to reduce the myocardial damage caused by sepsis in rats by decreasing the activity and expression of nitric oxide synthase and the production of NO [94]. Dong et al. [95] investigated the effects of cycloastragenol (CAG) on isoproterenol-induced myocardial fibrosis in mice. Furthermore, CAG was found to inhibit the transcription of NOX4 and iNOS as well as the phosphorylation of IκB-α and p65, which are key proteins in the NF-κB pathway. These findings suggest that CAG can alleviate myocardial fibrosis by suppressing oxidase activity, mitigating oxidative stress, and inhibiting the inflammatory response mediated by the NF-κB pathway.

### 5.4. Inhibit Lipid Peroxidation

Lipid peroxidation is a biochemical process in which lipids react with oxygen, forming peroxygen-free radicals and the subsequent generation of lipid hydrogen peroxide [96]. This process leads to the production of malondialdehyde (MDA), 4-hydroxynonenal, and other active aldehydes. These aldehydes can bind to proteins, causing protein inactivation, loss of function, changes in the physical properties of cell membranes, and disruption of redox homeostasis within cells [97]. Therefore, inhibiting lipid peroxidation plays a crucial role in maintaining redox homeostasis.

*Astragalus* saponins (PSMs) have been found to decrease the levels of MDA, which is a marker of lipid peroxidation, in both enzymatic- and non-enzymatic-induced lipid peroxidation models in spontaneously hypertensive and normal hypertensive rats. In the iron-induced lipid peroxidation model, the normotensive Wistar rats (NTRs) group experienced a 95% reduction in MDA production, while the spontaneously hypertensive rats (SHRs) group experienced a 25% reduction. In the in vitro NADPH-dependent CCl_4_-induced lipid peroxidation model, PSM reduced the MDA content by 55% in the NTRs group and 35% in the SHRs group [98]. This indicates that PSM effectively inhibits lipid peroxidation and reduces oxidative stress by decreasing MDA production. In a study conducted by Jing et al. [99], an *Astragalus* injection was administered to a rabbit model of an intestinal ischemia-reperfusion injury. The results showed that the *Astragalus* injection significantly reduced MDA content in the intestine, lungs, liver, kidney, and heart tissue. It also increased SOD activity and inhibited lipid peroxidation, protecting against intestinal ischemia-reperfusion injuries compared to the model group.

Que has been shown to reduce the activity of lipid oxygenase (5-LOX, 12-LOX) in plasma, thereby inhibiting lipid peroxidation and alleviating the inflammation caused by lipid peroxidation [100]. Additionally, Que has been found to significantly decrease the levels of MDA and increase the levels of glutathione (GSH). As an antioxidant, Que increases the levels of GSH and inhibits lipid peroxidation, ultimately playing a role in treating the kidney damage induced by gold nanoparticles in rats [101].

### 5.5. Chelating Metal Ions

Metals play a crucial role in various life activities and are essential for cell growth, differentiation, development, apoptosis, and immunity. Metals like iron, cobalt, and copper exhibit redox activity and participate in cyclic reactions with substrates to facilitate electron transfer in organisms. This process is vital for maintaining the body’s redox homeostasis. Recent research has shown that toxic metals can bind with proteins, DNA, and other biological macromolecules within cells, leading to oxidative degradation. This can ultimately result in gene mutation and protein degeneration, leading to the development of diseases such as cancer, Alzheimer’s disease, and diabetes [102]. However, the disruption of the homeostasis of redox-active metal ions can increase the production of reactive oxygen species, which can damage the cellular phospholipid bilayer and cause lipid peroxidation, leading to oxidative stress [103]. Therefore, chelating metal ions is an important mechanism to counteract oxidative stress.

*Astragalus* contains flavonoids, mainly Que, which have the ability to chelate metal ions. In a study using mice with an iron overload induced by ferrogluconate, Que was found to promote iron excretion by binding with the iron. This helped alleviate liver protein oxidation and lipid peroxidation caused by the iron overload [104]. In a study conducted by Li et al. [105], a primary hepatocyte oxidative damage model induced by Ferric-nitrilotriacetate was treated with Que. The study found that Que effectively inhibited abnormal labile iron pool (LIP) elevations and the oxidative damage induced by Ferric-nitrilotriacetate. Normal hepatocytes did not show any change in the LIP level, suggesting that the protective effect was associated with iron chelation activity. Furthermore, the primary metabolites of Que, ISO, and Tamarix exhibited a significant chelating force on iron, ferrous, and copper ions, which are influenced by the pH value of ferrous ions. As the pH level decreased from 7.5 to 4.5, the chelating force of these components on ferrous ions also decreased gradually [106]. The results suggest that both ISO and Tamarix can chelate iron and copper ions, potentially leading to an antioxidant effect through the chelation of metal ions. A study revealed that the chelating force of Que with iron highly depends on its chemical structure, with the 3-hydroxy-4-ketone (5-hydroxy-4) and catechol B rings as effective chelating sites. Under neutral conditions, Que exhibits similar activity to desferrioxamine and can chelate with iron in a 1:1 ratio, thus exerting an antioxidant effect [107].

### 5.6. Effects on Molecular Pathways

#### 5.6.1. Keap1/Nrf2 Signaling Pathway

The Keap1/Nrf2 pathway plays a significant role in regulating the body’s resistance to oxidative stress. Nrf2 is a key player in activating and up-regulating several antioxidant and detoxification genes, including SOD, GSH-Px, and HO-1. These genes express various enzymes that aid in detoxification and antioxidant processes. Conversely, Keap1 acts as a negative regulator of Nrf2 and serves as a sensor for oxidative and electrophilic stress. By regulating Nrf2’s activity, Keap1 plays an important role in combatting oxidative stress. Normally, Keap1, a component of E3 ubiquitin ligase, can bind to Nrf2 and hinder its activity and expression by promoting the ubiquitination of Nrf2 and subsequent degradation by the proteasome. However, when the body’s balance of redox reactions is disrupted, cysteine residues on Keap1 bind to electrophiles and ROS. This binding causes a conformational change in Keap1, allowing Nrf2 to avoid ubiquitination and translocate to the nucleus. Once in the nucleus, Nrf2 binds to the antioxidant response element (ARE) and promotes the transcription and expression of various antioxidant enzymes [108,109].

In a study conducted by Gao et al. [110], AS-IV intervention was administered to a tacrolimus-induced chronic renal toxicity mouse model for four weeks. The doses of AS-IV were administered orally at 10, 20, and 40 mg/kg/day. The results showed that AS-IV improved renal dysfunction and Tubulointerstitial fibrosis. It also increased the activities of SOD, CAT, and GSH-Px in kidney tissue. Additionally, AS-IV increased the accumulation of Nrf2 in the nucleus, the phosphorylation of p62, and the interaction with Keap1 in kidney tissue. In the HK-2 cell model induced by tacrolimus, AS-IV effectively decreased the levels of ROS and promoted the accumulation of Nrf2 in the nucleus. It also increased the levels of antioxidant factors downstream of Nrf2. However, the effects of AS-IV disappeared after the use of Nrf2 inhibitors. This suggests that AS-IV enhances the interaction between Keap1 and the phosphorylation of p62, leading to the accumulation of Nrf2 in the nucleus and the expression of antioxidant enzymes. Therefore, AS-IV has a protective effect on tacrolimus-induced chronic nephrotoxicity. Another study showed that when AS II is administered to streptozotocin-induced diabetic rats at doses of 3.2 and 6.4 mg/kg/day, it significantly relieved podocyte injuries. It also decreased Keap1 protein levels in rat kidney tissues. The expressions of Nrf2, PINK1, and Parkinson’s disease related to mitochondrial autophagy were increased. This suggests that AS II improves the ability of rats to resist oxidative stress by activating PINK1 and Nrf2 pathways, thus alleviating the damage of rat podocytes caused by diabetes [111].

Han et al. [112] conducted a study where they treated HK-2 cells damaged by oxalic acid with APSs of varying molecular weights. They found that all three different molecular weights of APSs were effective at repairing oxidative damage. Additionally, APS was observed to decrease ROS levels and increase the expression of Nrf2, SOD, and CAT. The mechanism of APS action may involve activating the Keap1-Nrf2 signaling pathway, up-regulating SOD and CAT expression, and ultimately reducing ROS levels. Furthermore, some APSs may promote the dissociation of Keap1/Nrf2 by binding to cysteine residues on Keap1, resulting in an antioxidant effect.

A recent study found that Que has the potential to reduce plaque area and lipid deposition, prevent macrophage pyroptosis, and enhance SOD and GSH levels. However, it was observed that these antioxidant enzyme effects diminished after treatment with an Nrf2 inhibitor [113]. These findings suggest that Que exerts anti-inflammatory and antioxidant effects by activating the Keap1/Nrf2 signaling pathway, which could have therapeutic implications for atherosclerosis.

In another study, it was discovered that ISO can potentially reduce the inflammatory response in mice with chronic obstructive pulmonary disease (COPD), resulting in improved lung function. Furthermore, ISO was observed to enhance the expression of Nrf2 and downstream antioxidant enzymes while decreasing the level of Keap1 and increasing the expression of the p62 protein [114]. This led to an augmented binding of p62 to Keap1, facilitating the dissociation of Nrf2 from Keap1 and enhancing the expression of antioxidant enzymes.

#### 5.6.2. PI3K/Akt Signaling Pathway

The PI3K/Akt pathway is an important intracellular signaling pathway that regulates various cellular processes, such as cell growth, survival, proliferation, differentiation, and metabolism [115]. In humans, there are three types of PI3K enzymes: Classes I, II, and III. Class I promotes the expression of the AKT kinase to regulate cell growth and proliferation and maintain cell homeostasis. Class II is responsible for regulating the endocytosis and exocytosis of phagosomes, as well as the K^+^ channel in immune cells. Class III promotes the formation of autophagosomes and the maturation of endocytosomes and lysosomes [116]. Akt, a serine/threonine protein kinase, plays a crucial role in inducing survival factors that inhibit apoptosis in a non-transcription-dependent manner [117]. Recent studies have also demonstrated the significant role of this pathway in the body’s resistance to oxidative stress. The activation of the PI3K/Akt pathway can protect cells from oxidative damage and apoptosis [118,119].

In a rat model of myocardial remodeling following myocardial infarction, the injection of *Astragalus* at doses of 1 mL/kg and 3 mL/kg, when administered intraperitoneally for 6 weeks, resulted in an increased survival time and survival rate in rats. *Astragalus* also showed improvements in myocardial injuries and fibrosis while inhibiting the production of MDA in the serum [120]. The up-regulation of endothelial nitric oxide synthase, PI3K, and the Akt protein expression suggests that *Astragalus* injections may have a therapeutic effect on myocardial remodeling after myocardial infarction. This effect is achieved by activating the PI3K/Akt pathway to resist oxidative stress.

In a study conducted by Cao et al. [121], mice with doxorubicin (DOX)-induced cardiotoxicity were administered APS orally at a dosage of 1.5 g/kg for a duration of 3 days. The findings of this research indicate that APS effectively suppresses the production of ROS, preventing the myocardial cell apoptosis induced by DOX. Additionally, APS was observed to induce the phosphorylation of Akt while inhibiting the phosphorylation of p38MARK. These results suggest that APS protects against DOX-induced oxidative stress and apoptosis by activating the PI3K/Akt pathway and inhibiting p38MARK phosphorylation.

In contrast to previous findings, Zheng et al. [122] conducted a study to investigate the effects of Que on rat models of premature ovarian failure (POF). The rats were administered Que at varying dosages of 25 mg/kg, 50 mg/kg, and 100 mg/kg, p.o., for a period of 30 days. The results of this study demonstrated that Que significantly improved the pathological status of the ovary in POF rats. This study further revealed that Que, a compound, can down-regulate the phosphorylation of the PI3K/Akt/FoxO3a signaling pathway and counteract oxidative stress in the ovarian tissue of rats with POF. This leads to a decrease in the content of MDA and an increase in the activities of GSH-Px, SOD, and CAT. Moreover, the expression of PI3K, AKT, and FoxO3a mRNA is inhibited, ultimately restoring ovarian function in POF rats.

Recent studies have demonstrated the close association between the PI3K/Akt signaling pathway and the Nrf2 pathway [123,124,125]. Yang et al. [126] conducted an experiment where H9c2 cardiomyocytes were subjected to hypoxia-reoxygenation-induced injuries and treated with 100 μM of AS-IV. The results showed that AS-IV significantly improved cell viability and SOD levels while reducing ROS, MDA, and LDH levels. Furthermore, AS-IV increased the expression of PI3K and p-Akt, as well as the nuclear expression of Nrf2 and the HO-1 protein in cells. However, these effects were partially reversed when PI3K-specific inhibitors were used, indicating that AS-IV exerts its antioxidant role by activating the PI3K/Akt/HO-1 signaling pathway. Another study [127] found that methylnissolin-3-O-β-D-glucopyranoside (MNG) in *Astragalus membranaceus* increased the expression of the antioxidant factors Nrf2, HO-1, and NQO1 in H_2_O_2_-treated EA.hy926 cells. However, the protective effect of MNG on EA.hy926 cells against oxidative damage through the Nrf2/HO-1 pathway, mediated by the PI3K/Akt pathway, was weakened after using PI3K inhibitors.

#### 5.6.3. NF-κB Signaling Pathway

The NF-κB transcription factor plays a crucial role in regulating cellular immunity and the inflammatory response. It is involved in various processes, including cell apoptosis, tumorigenicity, and inflammatory responses, making it an essential factor in cancer, autoimmune diseases [128], rheumatoid arthritis, asthma, chronic obstructive pulmonary disease, and other chronic inflammatory diseases [129]. Under normal conditions, the inhibitor of the NF-κB protein (IκB) binds to NF-κB and sequesters it in the cytoplasm to inhibit its activity and expression. However, when cells experience damage or stress, the IκB kinase (IKK) is activated, leading to the phosphorylation and degradation of the NF-κB-IκB complex. As a result, the NF-κB protein is released and transported to the nucleus [130], where it can bind to specific DNA sequences in promoters and enhancers. This binding ultimately regulates the gene expression in various cellular processes, such as cell proliferation, adhesion, apoptosis, inflammation, and stress responses [131].

In a study by Gao et al. [132], diabetic rats were treated with a combination of *Astragalus* and insulin. The results showed that rats in the insulin + *Astragalus* group had significantly lower levels of MDA and the NF-κB subunit expression in their kidneys compared to the insulin-only group. Additionally, SOD and GSH-Px activities significantly increased, and the serum creatinine and kidney weight ratio significantly decreased in the combination group. These findings suggest that administering a combination of *Astragalus* and insulin can effectively reduce the production of ROS by inhibiting the NF-κB pathway. This combination therapy may be protective in reducing renal oxidative damage in diabetic rats. Furthermore, Que has been shown to resist oxidative stress by inhibiting the activation of NF-κB and eliminating the IKK/NF-κB signal transduction pathway. As a result, it inhibits the overexpression of nitric oxide synthase, providing a protective effect against streptozocin-induced hepatic oxidative damage in diabetic rats, in addition to its other benefits [133].

In a research study, it was discovered that CA has a significant impact on reducing cerulein-induced pancreatic edema. Furthermore, it has the ability to suppress the levels of TNF-α, IL-6, IL-1β, and other inflammatory factors. Additionally, it can inhibit the activity of MPO while simultaneously increasing the activity of SOD [134]. CA acts as both an antioxidant and an anti-inflammatory agent by inhibiting the activation of NF-κB/p65 and the phosphorylation of IκBα, which are regulated by p38 and NF-κB signaling pathways. This inhibition ultimately leads to the alleviation of cerulein-induced acute pancreatitis.

In their study, Wang et al. [135] administered KAE (100 and 200 mg/kg/day, p.o., for two weeks) to a mouse model with cisplatin-induced renal toxicity. The results demonstrated that KAE had a protective effect on cisplatin-induced renal injuries by inhibiting lipid peroxidation, IKKα/β, and IκBα phosphorylation, as well as reducing nitric oxide synthase levels. KAE was found to regulate apoptosis, inflammation, and oxidative stress by inhibiting the NF-κB pathway and blocking the MAPK cascade, thereby playing a protective role in cisplatin-induced kidney injuries.

In a research study, it was discovered that AS II has the potential to enhance the condition of ulcerative colitis induced by dextran sulfate sodium (DSS) in mice. This improvement is attributed to the inhibition of the HIF-α/NF-κB pathway by AS II, which consequently reduces the expression of inflammatory factors and enhances the activity of SOD to combat oxidative stress [136].

#### 5.6.4. AMPK Signaling Pathway

AMP-activated protein kinase (AMPK) is a crucial regulatory factor that controls the synthesis and breakdown of energy and maintains mitochondrial homeostasis. It also significantly regulates cell growth, proliferation, differentiation, autophagy, and lipid metabolism. AMPK activation occurs when the ratio of ATP to AMP in cells changes, leading to a decrease in anabolism and an increase in catabolism, thereby regulating energy and mitochondrial homeostasis [137]. The maintenance of energy and mitochondrial homeostasis is closely associated with redox homeostasis. Recent studies have demonstrated that AMPK can enhance the expression of antioxidant enzymes and reduce the level of superoxide in order to counteract oxidative stress during stressful conditions, thus playing a vital role in maintaining the body’s redox homeostasis [138,139,140]. 

In a study conducted by Zhang et al. [141], APS treatment was administered to human vascular endothelial cells (HUVECs) induced by homocysteine (Hcy). The results demonstrated that APS improved the vitality of HUVECs, increased the expression of SOD1 and CAT mRNA, enhanced SOD activity, and reduced the level of MDA caused by Hcy. However, the protective effect of APS on HUVECs was weakened after adding an AMPK inhibitor, indicating that the activation of AMPK may play a crucial role in the protective effect of APS on HUVECs.

In a research study [142], it was found that Que can reduce sepsis caused by acute lung injury by activating the SIRT1/AMPK pathway. This activation leads to an improvement in SOD levels and a decrease in lipid peroxidation, ultimately inhibiting oxidative-stress-mediated endoplasmic reticulum stress and mitochondrial dysfunction. Zhang et al. [143] conducted a study where Que treatment was given to a rat model of diabetic atherosclerosis induced by a high-fat diet + streptozocin. The results showed that Que treatment reduced the level of pro-inflammatory factors in rat plasma, improved the activity of antioxidant enzymes such as SOD and GPX, decreased the level of MDA, and increased the expression of the SIRT1 protein when compared to the model group. When an AMPK inhibitor was used, Que’s promoting effect on SIRT1 protein expression disappeared. This suggests that Que may inhibit oxidative stress and inflammatory response by activating the AMPK/SIRT1/NF-κB signaling pathway, ultimately improving diabetic atherosclerosis in rats.

Dong et al. [144] conducted a study where they treated HepG2 cells induced by arachidonic acid + iron with IsoRN. The results showed that this treatment effectively inhibited liver cell death, reduced ROS production and GSH levels, improved mitochondrial function, and induced AMPK phosphorylation. These findings indicate that IsoRN has a protective effect against the oxidative damage induced by arachidonic acid and iron in hepatocytes. Furthermore, it was found that this protective effect is mediated by the activation of the AMPK signaling pathway.

Hu et al. [145] conducted a study to investigate the effects of CA treatment on the skeletal muscle atrophy caused by chronic kidney disease in a rat model. The rats were administered 15 mg/kg of CA intraperitoneally for a duration of 8 weeks. The results of this study demonstrated that CA improved renal function in rats compared to the model group. Additionally, CA reduced muscle atrophy and down-regulated the protein expression of APMK, SKP2, CARM1, and H3R17me2a. This study also observed an increase in SOD, GSH-Px, and CAT contents in the rat muscle tissue, while MDA levels decreased. Furthermore, the study found a reduction in the number of autophagosomes in rat muscles. These findings suggest that CA has the potential to alleviate oxidative stress and autophagy in skeletal muscles through the regulation of the AMPK/SKP2/CARM1 signaling pathway. Therefore, CA may serve as a promising therapeutic intervention for skeletal muscle atrophy resulting from chronic kidney disease.

#### 5.6.5. SIRT Signaling Pathway

In mammals, there are seven acetylases known as SIRT1-7. These SIRT family members are involved in the metabolism of mitochondrial oxidative droplets and play a role in inducing stress tolerance in cells. This coordination helps the body’s cellular response to stress [146]. Acetylases are essential components in various cellular activities, including the transcription, translation, protein stability, and regulation of oxidation levels [147]. Of particular importance are SIRT1, SIRT3, and SIRT5, which serve as key regulatory factors in mitochondria and play a significant role in oxidative stress responses and regulating mitochondrial energy metabolism [146].

In their study, Tang et al. [148] investigated the effects of AS-IV on retinal pigment epithelial cells induced by high sugar. This was achieved by reducing the expression of miRNA-138-5p, enhancing the activity of the SIRT1/Nrf2 signaling pathway, and ultimately inhibiting lipid peroxidation and an increase in ROS levels.

Yang et al. [149] conducted a study on rats with lung ischemia-reperfusion injuries. The rats were pretreated with KAE at different doses (12.5 mg/kg, 25 mg/kg, and 50 mg/kg, i.p., for 7 days). The study revealed that KAE significantly inhibited lung injuries caused by ischemia-reperfusion compared to the model group. The most effective dose was found at 50 mg/kg, which reduced MPO levels in lung tissue, lowered MDA levels, and increased SOD levels. Moreover, KAE reduced the expressions of the inflammatory cytokines IL-6 and TNF-α and inhibited the expressions of p-p65 and p65, which are the main regulatory proteins of the NF-κB pathway. The expression of HMGB 1 was observed to decrease, but this effect was diminished after administering the SIRT1 inhibitor EX 527. Furthermore, KAE was found to up-regulate the expression of SIRT1, suggesting that KAE’s protective effect on LIRI rats may be achieved by regulating the SIRT1/HMGB1/NF-κB axis to combat inflammation and oxidative stress.

According to a study [150], Que has been found to enhance mitochondrial function and maintain the mitochondrial homeostasis of cardiomyocytes by regulating SIRT1/TMBIM6. This regulation subsequently reduces oxidative damage and the apoptosis of human cardiomyocytes caused by hypoxia/reoxygenation. Another study [151] demonstrated that Que can regulate the SIRT3 protein to improve the levels of SOD2 and CAT in type 2 diabetes (T2DM) mice. As a result, it protects insulin β cells from apoptosis caused by oxidative stress.

In a study conducted by Oza et al. [152], T2DM rats were given varying doses of FMN (10 mg/kg, 20 mg/kg, and 40 mg/kg, e.g., for 16 weeks). The study observed that FMN had a positive effect on weight loss and renal hypertrophy in diabetic rats. Additionally, it reduced blood glucose levels, blood lipid levels, and insulin resistance in rats. The study also found that FMN decreased MDA levels and increased GSH levels, SOD, and CAT. Furthermore, it increased the expression of SIRT1. These findings suggest that FMN may have the potential to resist oxidative stress and reduce kidney injuries in diabetic nephropathy rats by increasing the expression of SIRT1. In a cell model of oxygen and glucose deprivation reperfusion (OGD/R), CG has been shown to alleviate OGD/R-induced cell damage, increase SOD activity, decrease ROS and MDA levels, and relieve the oxidative stress and apoptosis of cells. Additionally, CG has the potential to up-regulate the expression of SIRT1, FOXO1, and PGC-1α proteins. This finding suggests that CG may alleviate the oxidative damage caused by OGD/R through the regulation of the SIRT1/FOXO1/PGC-1α pathway [36].

In a study conducted by Zhai et al. [153], mice with DOX-induced cardiotoxicity were pretreated with CA at doses of 50 mg/kg and 100 mg/kg for 7 days. The results showed that CA effectively reduced MDA levels and increased the activities of GSH-Px, SOD, and CAT. Furthermore, CA inhibited the expression of IL-1β, TXNIP, caspase-1, and NLRP3 while increasing the expression of SIRT1 in a dose-dependent manner. These findings indicate that CA has the potential to improve myocardial injuries in mice. The protective effect of CA on DOX-induced cardiotoxicity was further confirmed through in vitro cell experiments. These findings suggest that CA may regulate the SIRT1-NLRP3 signaling pathway, thereby inhibiting oxidative stress and reducing cell apoptosis to protect against DOX-induced cardiotoxicity.

#### 5.6.6. PPAR-γ Signaling Pathway

As ligand-activated transcription factors belonging to the nuclear hormone receptor superfamily, peroxisome proliferator-activated receptors (PPARs) are categorized into three subtypes as follows: PPARα, PPARγ, and PPARβ/δ. PPARs are responsible for regulating glucose metabolism, energy balance, and fatty acid metabolism and play a significant role in maintaining energy homeostasis and metabolic function. Research has demonstrated that the PPAR signaling pathway also plays a role in regulating oxidative stress [38,154].

Zhang et al. [154] conducted a study where they treated human umbilical vein endothelial cells with 40 µM of FMN and a low-density lipoprotein (ox-LDL) for a duration of 24 h. The results of the study showed that FMN effectively reduced the endothelial injury induced by ox-LDL in these cells. The researchers also investigated the mechanisms underlying this effect and found that FMN improved the activity of SOD, reduced oxidative stress and inflammation induced by ox-LDL, and activated the PPAR-γ signaling pathway. These findings suggest that FMN may have a protective role in preventing atherosclerosis.

In a study conducted by Ma et al. [155], mice with LPS-induced lung injuries were treated with FMN (10 mg/kg and 20 mg/kg, 1 h before and 3 h after LPS instillation). The results showed that FMN inhibited MPO activity, improved the activity of SOD, alleviated the inflammatory response, and increased PPAR-γ expression. This suggests that FMN plays a protective role in acute lung injury by activating the PPAR-γ signaling pathway and inhibiting oxidative stress.

In their study, Liu et al. [156] investigated the effects of APS and targeted ultrasonic microvesicle destruction (UTMD) pretreatment on diabetic cardiomyopathy (DCM) rats. The findings demonstrated that the combination of UTMD and APS resulted in decreased MDA levels, an improvement in SOD and GSH-Px activities, and an increase in the expression of PPAR-γ. These results suggested that UTMD and APS could reduce oxidative stress by activating the PPAR-γ signaling pathway, thereby protecting against myocardial injury in DCM rats.

#### 5.6.7. Others

**Figure 2:**
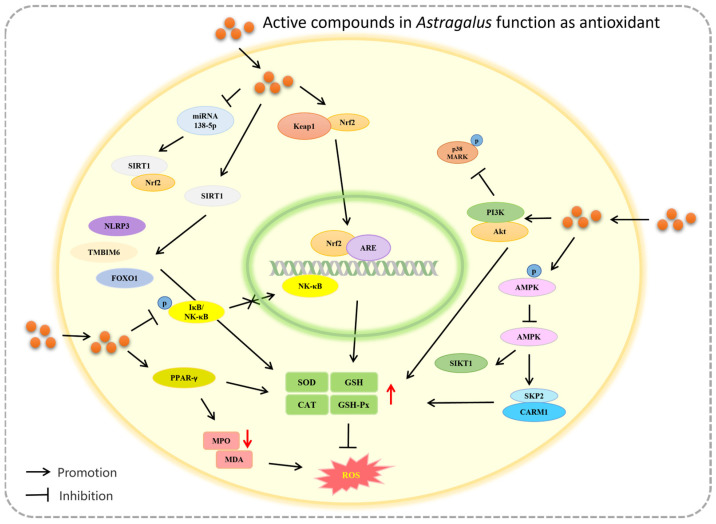
The mechanism of *Astragalus* against oxidative stress through molecular pathways.

Apart from the pathways mentioned earlier, *Astragalus* contains active ingredients that can also provide antioxidant effects through FoxO3a/Wnt2/β-catenin, JAK/STAT, and other signaling pathways. Ou et al. [157] conducted a study on a postmenopausal osteoporosis (PMOP) rat model treated with APS. The results demonstrated that APS could regulate the FoxO3a/Wnt2/β-catenin pathway, leading to an improvement in SOD and GSH-Px levels. These findings suggest that APS may have a therapeutic effect on osteoporosis caused by oxidative stress. Another study [158] discovered that ISO has the potential to enhance SOD activity, reduce MDA levels, and inhibit the H_2_O_2_-induced oxidative damage of HaCaT cells by regulating the Wnt signaling pathway. In a separate study [159], it was found that CA can inhibit liver fibrosis induced by CCl_4_ in mice. This effect was achieved through activating the JAK2/STAT3 signaling pathway, resistance to oxidative stress, and the regulation of the MMP-1/TIMP1 system. The mechanism of *Astragalus* against oxidative stress through molecular pathways is shown in Figure 2.

## 6. Future Prospects

While there has been extensive research on the antioxidant effects of the active components of *Astragali radix*, most of the research has been limited to animals and in vitro experiments. Clinical research is lacking, and there have been relatively few studies on the relationship between component structure and antioxidant activity. At present, the structural modification of the active components of *Astragalus* is still lacking, which may hinder clinical research. The antioxidant potential of *Astragali radix* is dependent on the absorption, distribution, metabolism, and bioavailability of each active component in vivo. Several active components of *Astragalus* have low bioavailability. In vitro and in vivo studies have primarily focused on APS, AS-IV, Que, Kae, and other components; however, there are limited reports on other saponins and some flavonoids. The future directions of pharmacological research on *Astragali radix* could take the following steps: (1) Further clinical studies could be carried out to explore the interaction of the active ingredients with antioxidant effects in *Astragali radix*, and the dosage range with significant effects should be determined. (2) Many antioxidant components in *Astragali radix* have the problem of low bioavailability, so it could be possible to develop effective antioxidant-derived drugs, nasal sprays, nanoparticles, and other drug delivery systems to increase their oral bioavailability. (3) There is a lack of relevant research on the structural modification of the active ingredients of *Astragali radix*. An in-depth exploration of the antioxidative action sites of the active ingredients is conducive to the development and application of derivative drugs, which provide new ideas for developing novel antioxidants.

## 7. Conclusions

Numerous studies in recent years have highlighted the crucial role of oxidative stress in various physiological and pathological processes, including aging, inflammation, neurodegenerative diseases, and cancer. Consequently, the search for new antioxidants has become a popular area of research.

TCM is applied under the guidance of TCM theory and has a long history of effective treatment. TCM is a valuable resource for new drug development due to its wide range of sources, low toxicity, and diverse active ingredients. *Astragali radix* is a classic representative of TCM and food with a long history of safe drug use and is highly advantageous. *Astragali radix* is a plant containing numerous bioactive substances, such as APS, AS-IV, CA, CG, Que, Kae, amino acids, and trace elements. Among these substances, polysaccharides, saponins, and flavonoids are natural antioxidants. It can resist oxidative stress by directly removing free radicals, improving the activity of antioxidant enzymes, inhibiting the activity of promoting enzymes, inhibiting lipid peroxidation, chelating metal ions, and regulating signal pathways, which provides insights into understanding the beneficial effects of *Astragali radix*, and sheds light when considering the further development of *Astragali radix* as a potential antioxidant action drug.

## Figures and Tables

**Table 1:** Summary of the studies reporting the main chemical components of *Astragali radix* and its antioxidant effects.

Category	Component	Structure	Dose	Exp. Model	Effect	Pharmacological Effects	References
Saponins	Astragaloside IV	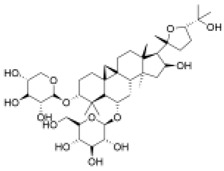	100 mg/kg	PD mouse	reduced the production of ROS and the accumulation of damaged mitochondria	anti-tumor [45], cardiovascular protection [46], kidney protection [47], neuroprotection [48]	[34]
Flavonones	Liquiritigenin	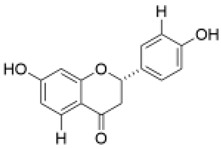	20 mg/kg, 40 mg/kg	Liver injury mice	increased GSH, SOD, and CAT levels in the serum of mice, inducing the levels of MDA and ROS	liver protection [35], anti-tumor [49], heart protection [50], and kidney protection [51]	[35]
Isoflavones	Calycosin-7-*O*-β-d glucoside	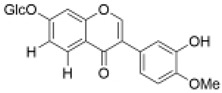	15 µg/mL	Oxygen–glucose deprivation/reperfusion (OGD/R) H22 model	improved cell viability and reduced oxidative stress	Liver protection [52] and cardiovascular protection [53]	[36]
Isoflavones	Calycosin	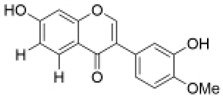	1, 10, 20 µM	PC12 cells	recovered the activity of the GSH, SOD/CAT content, and reduced the ROS content	kidney protection [54], cardiovascular protection [55], neuroprotection [56], lung protection [57]	[37]
Isoflavones	Ononin	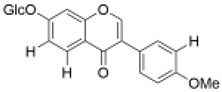	30 mg/kg	AD rats	improved the cognitive impairment and total antioxidant capacity	anti-inflammatory [58], anti-tumor [59], cardiac protection [60]	[38]
Isoflavones	Formononetin	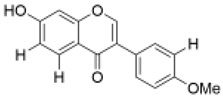	50 µM	SH-SY5Y cells	activated the PI3K/Akt-Nrf2 signaling pathway and decreased ROS levels	anti-inflammatory [61], anti-cancer [62], kidney protection [63]	[39]
Flavonols	Quercetin	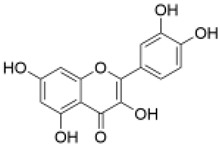	10, 20, 50, 100 µM	Lung epithelial A549 cells	suppressed NOX2 production and decreased ROS levels	anti-aging [64], cardiovascular protection [65]	[40]
Flavonols	Kaempferol	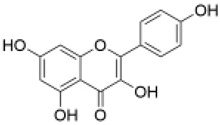	5, 10, 20 µM	Oleic acid-treated HepG2 cells	attenuated lipid accumulation and oxidative stress	cardiovascular protection [66], anti-cancer [67]	[41]
Flavonols	Isorhamnetin	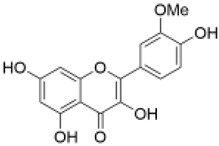	10 mg/kg	Diabetic mice	increased GSH levels and decreased MDA levels	anti-cancer [68], kidney protection [69], lung protection [70], liver protection [71]	[42]
Chalcones	Isoliquiritigenin	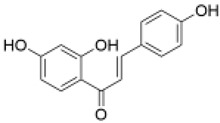	2, 4, 8 µM	PC12 cells	improved the activities of SOD, CAT, GSH-Px and inhibited the generation of ROS	kidney protection [72], liver protection [73], anti-inflammatory [74], antiviral [75], immune regulation [76], liver protection [77], heart protection [78]	[43]

## References

[1] Lyu Q., Zhao W.X., Wang S.J., Teng J.L., Xin D., Li J.X., Kong X.L. (2020). Effect of Astragali Radix Membranaceus in Promoting Blood Circulation and lts Modern Pharmacology Research. Chin. J. Exp. Tradit. Med. Formulae.

[2] Huang L.Q., He C.N., Ma P., Xiao P.G. (2022). Strategic Thinking on the Development of Food-Medicine Industry. Strateg. Study CAE.

[3] Mo W.Y., Lun C.H.I., Choi W.M., Man Y.B., Wong M.H. (2016). Enhancing growth and non-specific immunity of grass carp and Nile tilapia by incorporating Chinese herbs (*Astragalus membranaceus* and *Lycium barbarum*) into food waste based pellets. Environ. Pollut..

[4] Qiao Y., Liu C., Guo Y., Zhang W., Guo W., Oleksandr K., Wang Z. (2022). Polysaccharides derived from Astragalus membranaceus and Glycyrrhiza uralensis improve growth performance of broilers by enhancing intestinal health and modulating gut microbiota. Poult. Sci..

[5] Wu H.W., Li D.H., Song Q.J., Li G.F., Li X.W., Yang X.R., Li Y.F. (2022). Comparative study on chemical composition and in vitro anti-oxidant activity of Astragali Radix fresh-cut pieces and traditional pieces. Chin. Tradit. Herb. Drugs.

[6] Liu Y., Du J., Shen Y.H. (2017). Research Progress on Chemical Constituents and Pharmacology of 10 Kinds Medicinal Plants of Astragalus. Chin. J. Exp. Tradit. Med. Formulae.

[7] Wu D.X., Wang S.M., Chen S.J., Liu S.Y., Zhao H.X., Xiu Y. (2021). Rapid separation of astragalosides in Chinese patent medicines using inorganic nano-porous materials. Chin. J. Anal. Lab..

[8] Shi Y., Jia T.Y., Li X.R., Wei F., Ma S.C. (2022). Quantification of flavonoid compounds in Astragali Radix. Chin. J. Pharm. Anal..

[9] Fu J., Wang Z., Huang L., Zheng S., Wang D., Chen S., Zhang H., Yang S. (2014). Review of the botanical characteristics, phytochemistry, and pharmacology of *Astragalus membranaceus* (Huangqi). Phytother. Res..

[10] Guo Z., Lou Y., Kong M., Luo Q., Liu Z., Wu J. (2019). A Systematic Review of Phytochemistry, Pharmacology and Pharmacokinetics on Astragali Radix: Implications for Astragali Radix as a Personalized Medicine. Int. J. Mol. Sci..

[11] Zheng Y., Ren W., Zhang L., Zhang Y., Liu D., Liu Y. (2020). A Review of the Pharmacological Action of Astragalus Polysaccharide. Front. Pharmacol..

[12] Samuel A.O., Huang B.T., Chen Y., Guo F.X., Yang D.D., Jin J.Q. (2021). Antioxidant and antibacterial insights into the leaves, leaf tea and medicinal roots from *Astragalus membranaceus* (Fisch.) Bge. Sci. Rep..

[13] Yao J., Sun X.G., Dong R., Xie J.H., Wang Y.L., Yang X.N. (2021). Simultaneous quantitative analyses of six components in Astragalus membranaceus based on HPLC-CAD and quantitative analysis of multi- components with a single-marker. Acta Pharm. Sin..

[14] Gu Y., Wang G., Pan G., Fawcett J.P., A J., Sun J. (2004). Transport and bioavailability studies of astragaloside IV, an active ingredient in Radix Astragali. Basic Clin. Pharmacol. Toxicol..

[15] Zhang Q., Zhu L.L., Chen G.G., Du Y. (2007). Pharmacokinetics of astragaloside iv in beagle dogs. Eur. J. Drug Metab. Pharmacokinet..

[16] Singh S.P., Wahajuddin, Tewari D., Pradhan T., Jain G.K. (2011). PAMPA permeability, plasma protein binding, blood partition, pharmacokinetics and metabolism of formononetin, a methoxylated isoflavone. Food Chem. Toxicol..

[17] Luo L.Y., Fan M.X., Zhao H.Y., Li M.X., Wu X., Gao W.Y. (2018). Pharmacokinetics and Bioavailability of the Isoflavones Formononetin and Ononin and Their In Vitro Absorption in Ussing Chamber and Caco-2 Cell Models. J. Agric. Food Chem..

[18] Tao Y., Huang S., Yang G., Li W., Cai B. (2018). A simple and sensitive LC-MS/MS approach for simultaneous quantification of six bioactive compounds in rats following oral administration of aqueous extract and ultrafine powder of Astragalus propinquus: Application to a comparative pharmacokinetic study. J. Chromatogr. B.

[19] Shi J., Zheng H., Yu J., Zhu L., Yan T., Wu P., Lu L., Wang Y., Hu M., Liu Z. (2016). SGLT-1 Transport and Deglycosylation inside Intestinal Cells Are Key Steps in the Absorption and Disposition of Calycosin-7-O-β-d-Glucoside in Rats. Drug Metab. Dispos..

[20] Song S.S., Wang R.Y., Li Z.H., Yang Y., Wang T.T., Qing L.S., Luo P. (2023). Role of simulated in vitro gastrointestinal digestion on biotransformation and bioactivity of astragalosides from Radix Astragali. J. Pharm. Biomed. Anal..

[21] Shi Y., Liu W., Wang Y.L., Gong Z.P., Wang Q., Li Y.J., Jin Y., Yao X.Y. (2019). Study on the difference of astragaloside lV distribution between normal mice and db/db mice with type 2 diabetic nephropathy. Nat. Prod. Res. Dev..

[22] Sun W.X., Zhang Z.F., Xie J., He Y., Cheng Y., Ding L.S., Luo P., Qing L.S. (2019). Determination of a astragaloside IV derivative LS-102 in plasma by ultra-performance liquid chromatography-tandem mass spectrometry in dog plasma and its application in a pharmacokinetic study. Phytomedicine.

[23] Qing L.S., Chen T.B., Sun W.X., Chen L., Luo P., Zhang Z.F., Ding L.S. (2019). Pharmacokinetics Comparison, Intestinal Absorption and Acute Toxicity Assessment of a Novel Water-Soluble Astragaloside IV Derivative (Astragalosidic Acid, LS-102). Eur. J. Drug Metab. Pharmacokinet..

[24] Deng M., Chen H., Long J., Song J., Xie L., Li X. (2021). Calycosin: A Review of its Pharmacological Effects and Application Prospects. Expert Rev. Anti-Infect. Ther..

[25] de Boer V.C., Dihal A.A., van der Woude H., Arts I.C., Wolffram S., Alink G.M., Rietjens I.M., Keijer J., Hollman P.C. (2005). Tissue distribution of quercetin in rats and pigs. J. Nutr..

[26] Du Y., Wan H., Huang P., Yang J., He Y. (2022). A critical review of Astragalus polysaccharides: From therapeutic mechanisms to pharmaceutics. Biomed. Pharmacother..

[27] Liu P., Zhao H., Luo Y. (2017). Anti-Aging Implications of *Astragalus membranaceus* (Huangqi): A Well-Known Chinese Tonic. Aging Dis..

[28] Zhang J., Qiao Y., Li D., Hao S., Zhang F., Zhang X., Li A., Qin X. (2022). Aqueous Extract from *Astragalus membranaceus* Can Improve the Function Degradation and Delay Aging on Drosophila melanogaster Through Antioxidant Mechanism. Rejuvenation Res..

[29] Kan C.L., Li J.M., Hou J., Jing X.Y., Zhu Y.J., Zhang J.H., Guo Y.W., Chen X.R. (2019). *Astragalus membranaceus* and magnesium sulfate in gestational hypertension. Chin. J. Clin. Pharmacol..

[30] Yan X., Li X., Miao M. (2017). The intervention effect of different distribution ratio of Astragalus total saponins and curcumin on the DM rats model. Saudi Pharm. J..

[31] Ou Y.Y., Chen X.R., Zheng L.X., Li L., Li W.F. (2017). *Astragalus membranaceus* inhibits bleomycin-induced pulmonary fibrosis in mice by antioxidation. Chin. J. Pathophysiol..

[32] Adesso S., Russo R., Quaroni A., Autore G., Marzocco S. (2018). *Astragalus membranaceus* Extract Attenuates Inflammation and Oxidative Stress in Intestinal Epithelial Cells via NF-κB Activation and Nrf2 Response. Int. J. Mol. Sci..

[33] Sheng Z., Jiang Y., Liu J., Yang B. (2021). UHPLC-MS/MS Analysis on Flavonoids Composition in *Astragalus membranaceus* and Their Antioxidant Activity. Antioxidants.

[34] Xia M.L., Xie X.H., Ding J.H., Du R.H., Hu G. (2020). Astragaloside IV inhibits astrocyte senescence: Implication in Parkinson’s disease. J. Neuroinflamm..

[35] Zhang M., Xue Y., Zheng B., Li L., Chu X., Zhao Y., Wu Y., Zhang J., Han X., Wu Z. (2021). Liquiritigenin protects against arsenic trioxide-induced liver injury by inhibiting oxidative stress and enhancing mTOR-mediated autophagy. Biomed. Pharmacother..

[36] Yan X., Yu A., Zheng H., Wang S., He Y., Wang L. (2019). Calycosin-7-O-β-D-glucoside Attenuates OGD/R-Induced Damage by Preventing Oxidative Stress and Neuronal Apoptosis via the SIRT1/FOXO1/PGC-1α Pathway in HT22 Cells. Neural Plast..

[37] Pan Q., Ban Y., Khan S. (2021). Antioxidant activity of calycosin against α-synuclein amyloid fibrils-induced oxidative stress in neural-like cells as a model of preventive care studies in Parkinson’s disease. Int. J. Biol. Macromol..

[38] Chen X., Zhang M., Ahmed M., Surapaneni K.M., Veeraraghavan V.P., Arulselvan P. (2021). Neuroprotective effects of ononin against the aluminium chloride-induced Alzheimer’s disease in rats. Saudi J. Biol. Sci..

[39] Sugimoto M., Ko R., Goshima H., Koike A., Shibano M., Fujimori K. (2021). Formononetin attenuates H(2)O(2)-induced cell death through decreasing ROS level by PI3K/Akt-Nrf2-activated antioxidant gene expression and suppressing MAPK-regulated apoptosis in neuronal SH-SY5Y cells. Neurotoxicology.

[40] Sul O.J., Ra S.W. (2021). Quercetin Prevents LPS-Induced Oxidative Stress and Inflammation by Modulating NOX2/ROS/NF-kB in Lung Epithelial Cells. Molecules.

[41] Tie F., Ding J., Hu N., Dong Q., Chen Z., Wang H. (2021). Kaempferol and Kaempferide Attenuate Oleic Acid-Induced Lipid Accumulation and Oxidative Stress in HepG2 Cells. Int. J. Mol. Sci..

[42] Alqudah A., Qnais E.Y., Wedyan M.A., Altaber S., Bseiso Y., Oqal M., AbuDalo R., Alrosan K., Alrosan A.Z., Bani Melhim S. (2023). Isorhamnetin Reduces Glucose Level, Inflammation, and Oxidative Stress in High-Fat Diet/Streptozotocin Diabetic Mice Model. Molecules.

[43] Shi D., Yang J., Jiang Y., Wen L., Wang Z., Yang B. (2020). The antioxidant activity and neuroprotective mechanism of isoliquiritigenin. Free Radic. Biol. Med..

[44] Li M., Xu J., Wang Y., Du X., Zhang T., Chen Y. (2022). Astragaloside A Protects Against Photoreceptor Degeneration in Part Through Suppressing Oxidative Stress and DNA Damage-Induced Necroptosis and Inflammation in the Retina. J. Inflamm. Res..

[45] Xu F., Cui W.Q., Wei Y., Cui J., Qiu J., Hu L.L., Gong W.Y., Dong J.C., Liu B.J. (2018). Astragaloside IV inhibits lung cancer progression and metastasis by modulating macrophage polarization through AMPK signaling. J. Exp. Clin. Cancer Res..

[46] Meng P., Yang R., Jiang F., Guo J., Lu X., Yang T., He Q. (2021). Molecular Mechanism of Astragaloside IV in Improving Endothelial Dysfunction of Cardiovascular Diseases Mediated by Oxidative Stress. Oxidative Med. Cell. Longev..

[47] Zhou W., Chen Y., Zhang X. (2017). Astragaloside IV Alleviates Lipopolysaccharide-Induced Acute Kidney Injury Through Down-Regulating Cytokines, CCR5 and p-ERK, and Elevating Anti-Oxidative Ability. Med. Sci. Monit..

[48] Yao M., Zhang L., Wang L. (2023). Astragaloside IV: A promising natural neuroprotective agent for neurological disorders. Biomed. Pharmacother..

[49] Liu H., Yao Z., Sun M., Zhang C., Huang Y.Y., Luo H.B., Wu D., Zheng X. (2023). Inhibition of AKR1Cs by liquiritigenin and the structural basis. Chem.-Biol. Interact..

[50] Zhang M., Qi J., He Q., Ma D., Li J., Chu X., Zuo S., Zhang Y., Li L., Chu L. (2022). Liquiritigenin protects against myocardial ischemic by inhibiting oxidative stress, apoptosis, and L-type Ca(2+) channels. Phytother. Res..

[51] Zhou M., Dai Y., Ma Y., Yan Y., Hua M., Gao Q., Geng X., Zhou Q. (2022). Protective Effects of Liquiritigenin against Cisplatin-Induced Nephrotoxicity via NRF2/SIRT3-Mediated Improvement of Mitochondrial Function. Molecules.

[52] Xu W., Zhou F., Zhu Q., Bai M., Luo T., Zhou L., Deng R. (2022). Calycosin-7-O-β-D-glucoside attenuates palmitate-induced lipid accumulation in hepatocytes through AMPK activation. Eur. J. Pharmacol..

[53] Tsai C.C., Wu H.H., Chang C.P., Lin C.H., Yang H.H. (2019). Calycosin-7-O-β-D-glucoside reduces myocardial injury in heat stroke rats. J. Formos. Med. Assoc..

[54] Chen G., Xu H., Xu T., Ding W., Zhang G., Hua Y., Wu Y., Han X., Xie L., Liu B. (2022). Calycosin reduces myocardial fibrosis and improves cardiac function in post-myocardial infarction mice by suppressing TGFBR1 signaling pathways. Phytomedicine.

[55] Huang D., Shen P., Wang C., Gao J., Ye C., Wu F. (2022). Calycosin plays a protective role in diabetic kidney disease through the regulation of ferroptosis. Pharm. Biol..

[56] Pan L., Zhang X.F., Wei W.S., Zhang J., Li Z.Z. (2020). The cardiovascular protective effect and mechanism of calycosin and its derivatives. Chin. J. Nat. Med..

[57] Li J., Peng J., Tan X. (2022). Calycosin alleviates hyperbilirubin nerve injury in Ugt1(-/-) mice by inhibiting oxidative stress, apoptosis, and mitochondrial function. Acta Histochem..

[58] Liu H., Bai X., Wei W., Li Z., Zhang Z., Tan W., Wei B., Zhao H., Jiao Y. (2022). Calycosin Ameliorates Bleomycin-Induced Pulmonary Fibrosis via Suppressing Oxidative Stress, Apoptosis, and Enhancing Autophagy. Evid.-Based Complement. Altern. Med..

[59] Yu T., Lu X., Liang Y., Yang L., Yin Y., Chen H. (2023). Ononin alleviates DSS-induced colitis through inhibiting NLRP3 inflammasome via triggering mitophagy. Immun. Inflamm. Dis..

[60] Ye B., Ma J., Li Z., Li Y., Han X. (2022). Ononin Shows Anticancer Activity Against Laryngeal Cancer via the Inhibition of ERK/JNK/p38 Signaling Pathway. Front. Oncol..

[61] Pan R., Zhuang Q., Wang J. (2021). Ononin alleviates H(2)O(2)-induced cardiomyocyte apoptosis and improves cardiac function by activating the AMPK/mTOR/autophagy pathway. Exp. Ther. Med..

[62] Yu L., Zhang Y., Chen Q., He Y., Zhou H., Wan H., Yang J. (2022). Formononetin protects against inflammation associated with cerebral ischemia-reperfusion injury in rats by targeting the JAK2/STAT3 signaling pathway. Biomed. Pharmacother..

[63] Jiang D., Rasul A., Batool R., Sarfraz I., Hussain G., Mateen Tahir M., Qin T., Selamoglu Z., Ali M., Li J. (2019). Potential Anticancer Properties and Mechanisms of Action of Formononetin. BioMed Res. Int..

[64] Hao Y., Miao J., Liu W., Peng L., Chen Y., Zhong Q. (2021). Formononetin protects against cisplatin-induced acute kidney injury through activation of the PPARα/Nrf2/HO-1/NQO1 pathway. Int. J. Mol. Med..

[65] Özsoy Gökbilen S., Becer E., Vatansever H.S. (2022). Senescence-mediated anticancer effects of quercetin. Nutr. Res..

[66] Dabeek W.M., Marra M.V. (2019). Dietary Quercetin and Kaempferol: Bioavailability and Potential Cardiovascular-Related Bioactivity in Humans. Nutrients.

[67] Imran M., Salehi B., Sharifi-Rad J., Aslam Gondal T., Saeed F., Imran A., Shahbaz M., Tsouh Fokou P.V., Umair Arshad M., Khan H. (2019). Kaempferol: A Key Emphasis to Its Anticancer Potential. Molecules.

[68] Sarkar S., Das A.K., Bhattacharya S., Gachhui R., Sil P.C. (2023). Isorhamnetin exerts anti-tumor activity in DEN + CCl(4)-induced HCC mice. Med. Oncol..

[69] Wang L., Xie Y., Xiao B., He X., Ying G., Zha H., Yang C., Jin X., Li G., Ping L. (2024). Isorhamnetin alleviates cisplatin-induced acute kidney injury via enhancing fatty acid oxidation. Free Radic. Biol. Med..

[70] Yang B., Ma L., Wei Y., Cui Y., Li X., Wei Y., Zhang S., Zhang L., Zhou H., Wang G. (2022). Isorhamnetin alleviates lipopolysaccharide-induced acute lung injury by inhibiting mTOR signaling pathway. Immunopharmacol. Immunotoxicol..

[71] Rousta A.M., Mirahmadi S.M., Shahmohammadi A., Mehrabi Z., Fallah S., Baluchnejadmojarad T., Roghani M. (2022). Therapeutic Potential of Isorhamnetin following Acetaminophen-Induced Hepatotoxicity through Targeting NLRP3/NF-κB/Nrf2. Drug Res..

[72] Tang Y., Luo H., Xiao Q., Li L., Zhong X., Zhang J., Wang F., Li G., Wang L., Li Y. (2021). Isoliquiritigenin attenuates septic acute kidney injury by regulating ferritinophagy-mediated ferroptosis. Ren. Fail..

[73] Huang S., Wang Y., Xie S., Lai Y., Mo C., Zeng T., Kuang S., Zhou C., Zeng Z., Chen Y. (2022). Isoliquiritigenin alleviates liver fibrosis through caveolin-1-mediated hepatic stellate cells ferroptosis in zebrafish and mice. Phytomedicine.

[74] Chen Z., Ding W., Yang X., Lu T., Liu Y. (2024). Isoliquiritigenin, a potential therapeutic agent for treatment of inflammation-associated diseases. J. Ethnopharmacol..

[75] Wang H., Jia X., Zhang M., Cheng C., Liang X., Wang X., Xie F., Wang J., Yu Y., He Y. (2023). Isoliquiritigenin inhibits virus replication and virus-mediated inflammation via NRF2 signaling. Phytomedicine.

[76] Su Y., Fang L., Zhong K., Wang T., Bao M., Zhou T., Zhu Y. (2023). Isoliquiritigenin induces oxidative stress and immune response in zebrafish embryos. Environ. Toxicol..

[77] Ni B., Liu Y., Gao X., Cai M., Fu J., Yin X., Ni J., Dong X. (2022). Isoliquiritigenin attenuates emodin-induced hepatotoxicity in vivo and in vitro through Nrf2 pathway. Comp. Biochem. Physiol. C-Toxicol. Pharmacol..

[78] Yao D., Shi B., Wang S., Bao L., Tan M., Shen H., Zhang Z., Pan X., Yang Y., Wu Y. (2022). Isoliquiritigenin Ameliorates Ischemia-Induced Myocardial Injury via Modulating the Nrf2/HO-1 Pathway in Mice. Drug Des. Dev. Ther..

[79] Ienco E.C., LoGerfo A., Carlesi C., Orsucci D., Ricci G., Mancuso M., Siciliano G. (2011). Oxidative stress treatment for clinical trials in neurodegenerative diseases. J. Alzheimer’s Dis..

[80] Venkataraman K., Khurana S., Tai T.C. (2013). Oxidative stress in aging—Matters of the heart and mind. Int. J. Mol. Sci..

[81] Bhatti J.S., Bhatti G.K., Reddy P.H. (2017). Mitochondrial dysfunction and oxidative stress in metabolic disorders—A step towards mitochondria based therapeutic strategies. Biochim. Et Biophys. Acta-Mol. Basis Dis..

[82] Carocho M., Ferreira I.C. (2013). A review on *Antioxidants*, prooxidants and related controversy: Natural and synthetic compounds, screening and analysis methodologies and future perspectives. Food Chem. Toxicol..

[83] Salim S. (2017). Oxidative Stress and the Central Nervous System. J. Pharmacol. Exp. Ther..

[84] Liu Y., Liu F., Yang Y., Li D., Lv J., Ou Y., Sun F., Chen J., Shi Y., Xia P. (2014). Astragalus polysaccharide ameliorates ionizing radiation-induced oxidative stress in mice. Int. J. Biol. Macromol..

[85] Zhou J., Zhang N., Zhao L., Wu W., Zhang L., Zhou F., Li J. (2021). Astragalus Polysaccharides and Saponins Alleviate Liver Injury and Regulate Gut Microbiota in Alcohol Liver Disease Mice. Foods.

[86] Xing J.W., CHEN H., YU Z.H., Zhang B.P. (2022). Effect of Astragalus Polysaccharides on oxidative stress in mice bearing ascites tumor. Chin. J. Clin. Pharmacol..

[87] Ma L.L., Li H., Sun L.M., Dong Y., Yi B.H. (2019). Effects of Astragali Radix total flavonoids on oxidative stress, inflammation and apoptosisof rats with cerebral ischemia-reperfusion injury. Chin. Tradit. Pat. Med..

[88] Okumoto K., Tamura S., Honsho M., Fujiki Y. (2020). Peroxisome: Metabolic Functions and Biogenesis. Adv. Exp. Med. Biol..

[89] Zou H.L., Chen M., WU Q.P., Meng L.J., Liu B.L., Chen K. (2023). Extraction process optimization and enzyme kinetics for active components inhibitingxanthine oxidase from Smilax glabra. Chin. Tradit. Pat. Med..

[90] Li J., Jiang L.L., Li M.X., Deng F., Xu S.Y., Xue X., Hu R.P., Xue H.T. (2021). Inhibitory Effects of Astragalus Polysaccharide on Activity of Xanthine Oxidase. J. Food Sci. Biotechnol..

[91] Lin J., Fang L., Li H., Li Z., Lyu L., Wang H., Xiao J. (2019). Astragaloside IV alleviates doxorubicin induced cardiomyopathy by inhibiting NADPH oxidase derived oxidative stress. Eur. J. Pharmacol..

[92] Jimenez R., Lopez-Sepulveda R., Romero M., Toral M., Cogolludo A., Perez-Vizcaino F., Duarte J. (2015). Quercetin and its metabolites inhibit the membrane NADPH oxidase activity in vascular smooth muscle cells from normotensive and spontaneously hypertensive rats. Food Funct..

[93] Ye L.H., Yan M.Z., Kong L.T., He M., Chang Q. (2014). ln vitro Inhibition of Quercetin and lts Glycosides on P450 Enzyme Activities. Chin. Pharm. J..

[94] Zhang C., Huang L., QU S.L., Tang Z.H., Wei X. (2011). Quercetin inhibits inducible NO synthase overexpression against sepsis-induced myocardial depression. Chin. Pharmacol. Bull..

[95] Dong X., Fan H.W., Hu B.X., Zhang Y., Zhang Z. (2020). Effects of cycloastragenol on cardiac fibrosis induced by isoproterenol in mice. Chin. J. Pathophysiol..

[96] Conrad M., Kagan V.E., Bayir H., Pagnussat G.C., Head B., Traber M.G., Stockwell B.R. (2018). Regulation of lipid peroxidation and ferroptosis in diverse species. Genes Dev..

[97] Pisoschi A.M., Pop A. (2015). The role of *Antioxidants* in the chemistry of oxidative stress: A review. Eur. J. Med. Chem..

[98] Simeonova R.L., Vitcheva V.B., Kondeva-Burdina M.S., Krasteva I.N., Nikolov S.D., Mitcheva M.K. (2010). Effect of purified saponin mixture from Astragalus corniculatus on enzyme- and non-enzyme-induced lipid peroxidation in liver microsomes from spontaneously hypertensive rats and normotensive rats. Phytomedicine.

[99] Jing Y.L., Wang Y.L., Wang X.J., Zhang W., Duan G.X., Zhang Y.B., Zhao C.X. (2009). Effect of Astragalus on lipid peroxidation injury with ischemia/reperfusion (I/R) of intestinal and the pathogenesis. Chin. J. Appl. Physiol..

[100] Bhaskar S., Kumar K.S., Krishnan K., Antony H. (2013). Quercetin alleviates hypercholesterolemic diet induced inflammation during progression and regression of atherosclerosis in rabbits. Nutrition.

[101] Abdelhalim M.A.K., Qaid H.A., Al-Mohy Y., Al-Ayed M.S. (2018). Effects of quercetin and arginine on the nephrotoxicity and lipid peroxidation induced by gold nanoparticles in vivo. Int. J. Nanomed..

[102] Jomova K., Valko M. (2011). Advances in metal-induced oxidative stress and human disease. Toxicology.

[103] Valko M., Jomova K., Rhodes C.J., Kuča K., Musílek K. (2016). Redox- and non-redox-metal-induced formation of free radicals and their role in human disease. Arch. Toxicol..

[104] Zhang Y., Li H., Zhao Y., Gao Z. (2006). Dietary supplementation of baicalin and quercetin attenuates iron overload induced mouse liver injury. Eur. J. Pharmacol..

[105] Li Y., Deng Y., Tang Y., Yu H., Gao C., Liu L., Liu L., Yao P. (2014). Quercetin protects rat hepatocytes from oxidative damage induced by ethanol and iron by maintaining intercellular liable iron pool. Hum. Exp. Toxicol..

[106] Lomozová Z., Catapano M.C., Hrubša M., Karlíčková J., Macáková K., Kučera R., Mladěnka P. (2021). Chelation of Iron and Copper by Quercetin B-Ring Methyl Metabolites, Isorhamnetin and Tamarixetin, and Their Effect on Metal-Based Fenton Chemistry. J. Agric. Food Chem..

[107] Mladěnka P., Macáková K., Filipský T., Zatloukalová L., Jahodář L., Bovicelli P., Silvestri I.P., Hrdina R., Saso L. (2011). In vitro analysis of iron chelating activity of flavonoids. J. Inorg. Biochem..

[108] Yu C., Xiao J.H. (2021). The Keap1-Nrf2 System: A Mediator between Oxidative Stress and Aging. Oxidative Med. Cell. Longev..

[109] Yao J., Wu P.A., LI Y., Li Y.F., Liu D.L., Liu X.F. (2019). Research progress of small molecule activators in Keap1-Nrf2-ARE signaling pathway. Chin. Pharmacol. Bull..

[110] Gao P., Du X., Liu L., Xu H., Liu M., Guan X., Zhang C. (2020). Astragaloside IV Alleviates Tacrolimus-Induced Chronic Nephrotoxicity via p62-Keap1-Nrf2 Pathway. Front. Pharmacol..

[111] Su J., Gao C., Xie L., Fan Y., Shen Y., Huang Q., Wang N., Xu Y., Yang N., Gui D. (2021). Astragaloside II Ameliorated Podocyte Injury and Mitochondrial Dysfunction in Streptozotocin-Induced Diabetic Rats. Front. Pharmacol..

[112] Han J., Guo D., Sun X.Y., Wang J.M., Ouyang J.M., Gui B.S. (2019). Repair Effects of Astragalus Polysaccharides with Different Molecular Weights on Oxidatively Damaged HK-2 Cells. Sci. Rep..

[113] Luo X., Weng X., Bao X., Bai X., Lv Y., Zhang S., Chen Y., Zhao C., Zeng M., Huang J. (2022). A novel anti-atherosclerotic mechanism of quercetin: Competitive binding to KEAP1 via Arg483 to inhibit macrophage pyroptosis. Redox Biol..

[114] Xu Y., Li J., Lin Z., Liang W., Qin L., Ding J., Chen S., Zhou L. (2022). Isorhamnetin Alleviates Airway Inflammation by Regulating the Nrf2/Keap1 Pathway in a Mouse Model of COPD. Front. Pharmacol..

[115] Noorolyai S., Shajari N., Baghbani E., Sadreddini S., Baradaran B. (2019). The relation between PI3K/AKT signalling pathway and cancer. Gene.

[116] Jafari M., Ghadami E., Dadkhah T., Akhavan-Niaki H. (2019). PI3k/AKT signaling pathway: Erythropoiesis and beyond. J. Cell. Physiol..

[117] Revathidevi S., Munirajan A.K. (2019). Akt in cancer: Mediator and more. Semin. Cancer Biol..

[118] Ma Z., Zhang W., Wu Y., Zhang M., Wang L., Wang Y., Wang Y., Liu W. (2021). Cyclophilin A inhibits A549 cell oxidative stress and apoptosis by modulating the PI3K/Akt/mTOR signaling pathway. Biosci. Rep..

[119] Zhang B.H., Liu H., Yuan Y., Weng X.D., Du Y., Chen H., Chen Z.Y., Wang L., Liu X.H. (2021). Knockdown of TRIM8 Protects HK-2 Cells Against Hypoxia/Reoxygenation-Induced Injury by Inhibiting Oxidative Stress-Mediated Apoptosis and Pyroptosis via PI3K/Akt Signal Pathway. Drug Des. Dev. Ther..

[120] Liu F., Cui X.M., Xu J., Yu Z.Y., Guan F.Y. (2019). Protective effect of astragalus injection in cardiac remodeling after acute myocardial infarction via PI3K/AKT pathway activation. Chin. J. Comp. Med..

[121] Cao Y., Ruan Y., Shen T., Huang X., Li M., Yu W., Zhu Y., Man Y., Wang S., Li J. (2014). Astragalus polysaccharide suppresses doxorubicin-induced cardiotoxicity by regulating the PI3k/Akt and p38MAPK pathways. Oxidative Med. Cell. Longev..

[122] Zheng S., Ma M., Chen Y., Li M. (2022). Effects of quercetin on ovarian function and regulation of the ovarian PI3K/Akt/FoxO3a signalling pathway and oxidative stress in a rat model of cyclophosphamide-induced premature ovarian failure. Basic Clin. Pharmacol. Toxicol..

[123] Zhuang Y., Wu H., Wang X., He J., He S., Yin Y. (2019). Resveratrol Attenuates Oxidative Stress-Induced Intestinal Barrier Injury through PI3K/Akt-Mediated Nrf2 Signaling Pathway. Oxidative Med. Cell. Longev..

[124] Li J., Wang T., Liu P., Yang F., Wang X., Zheng W., Sun W. (2021). Hesperetin ameliorates hepatic oxidative stress and inflammation via the PI3K/AKT-Nrf2-ARE pathway in oleic acid-induced HepG2 cells and a rat model of high-fat diet-induced NAFLD. Food Funct..

[125] Li S.T., Dai Q., Zhang S.X., Liu Y.J., Yu Q.Q., Tan F., Lu S.H., Wang Q., Chen J.W., Huang H.Q. (2018). Ulinastatin attenuates LPS-induced inflammation in mouse macrophage RAW264.7 cells by inhibiting the JNK/NF-κB signaling pathway and activating the PI3K/Akt/Nrf2 pathway. Acta Pharmacol. Sin..

[126] Yang P., Zhou Y., Xia Q., Yao L., Chang X. (2019). Astragaloside IV Regulates the PI3K/Akt/HO-1 Signaling Pathway and Inhibits H9c2 Cardiomyocyte Injury Induced by Hypoxia-Reoxygenation. Biol. Pharm. Bull..

[127] Wu X., Xu J., Cai Y., Yang Y., Liu Y., Cao S. (2021). Cytoprotection against Oxidative Stress by Methylnissolin-3-O-β-d-glucopyranoside from Astragalus membranaceus Mainly via the Activation of the Nrf2/HO-1 Pathway. Molecules.

[128] Barnabei L., Laplantine E., Mbongo W., Rieux-Laucat F., Weil R. (2021). NF-κB: At the Borders of Autoimmunity and Inflammation. Front. Immunol..

[129] Lawrence T. (2009). The nuclear factor NF-kappaB pathway in inflammation. Cold Spring Harb. Perspect. Biol..

[130] Napetschnig J., Wu H. (2013). Molecular basis of NF-κB signaling. Annu. Rev. Biophys..

[131] Wu M., Bian Q., Liu Y., Fernandes A.F., Taylor A., Pereira P., Shang F. (2009). Sustained oxidative stress inhibits NF-kappaB activation partially via inactivating the proteasome. Free Radic. Biol. Med..

[132] Gao Y., Zhang R.R., Li J.H., Ren M., Ren Z.X., Shi J.H., Pan Q.Z., Ren S.P. (2012). Radix Astragali lowers kidney oxidative stress in diabetic rats treated with insulin. Endocrine.

[133] Dias A.S., Porawski M., Alonso M., Marroni N., Collado P.S., González-Gallego J. (2005). Quercetin decreases oxidative stress, NF-kappaB activation, and iNOS overexpression in liver of streptozotocin-induced diabetic rats. J. Nutr..

[134] Ma R., Yuan F., Wang S., Liu Y., Fan T., Wang F. (2018). Calycosin alleviates cerulein-induced acute pancreatitis by inhibiting the inflammatory response and oxidative stress via the p38 MAPK and NF-κB signal pathways in mice. Biomed. Pharmacother..

[135] Wang Z., Sun W., Sun X., Wang Y., Zhou M. (2020). Kaempferol ameliorates Cisplatin induced nephrotoxicity by modulating oxidative stress, inflammation and apoptosis via ERK and NF-κB pathways. AMB Express.

[136] Qiao C., Wan J., Zhang L., Luo B., Liu P., Di A., Gao H., Sun X., Zhao G. (2019). Astragaloside II alleviates the symptoms of experimental ulcerative colitis in vitro and in vivo. Am. J. Transl. Res..

[137] Herzig S., Shaw R.J. (2018). AMPK: Guardian of metabolism and mitochondrial homeostasis. Nat. Rev. Mol. Cell Biol..

[138] Sid B., Verrax J., Calderon P.B. (2013). Role of AMPK activation in oxidative cell damage: Implications for alcohol-induced liver disease. Biochem. Pharmacol..

[139] Xu W., Zhao T., Xiao H. (2020). The Implication of Oxidative Stress and AMPK-Nrf2 Antioxidative Signaling in Pneumonia Pathogenesis. Front. Endocrinol..

[140] Jeon S.M. (2016). Regulation and function of AMPK in physiology and diseases. Exp. Mol. Med..

[141] Zhang L., Song J., Sun Y.L., Zheng Y.J., Lin D.J., Fang Z.G. (2018). Astragalus polysaccharides attenuate endothelial cell injury caused by homocysteine via AMPK pathway. Chin. J. Pathophysiol..

[142] Sang A., Wang Y., Wang S., Wang Q., Wang X., Li X., Song X. (2022). Quercetin attenuates sepsis-induced acute lung injury via suppressing oxidative stress-mediated ER stress through activation of SIRT1/AMPK pathways. Cell. Signal..

[143] Zhang F., Feng J., Zhang J., Kang X., Qian D. (2020). Quercetin modulates AMPK/SIRT1/NF-κB signaling to inhibit inflammatory/oxidative stress responses in diabetic high fat diet-induced atherosclerosis in the rat carotid artery. Exp. Ther. Med..

[144] Dong G.Z., Lee J.H., Ki S.H., Yang J.H., Cho I.J., Kang S.H., Zhao R.J., Kim S.C., Kim Y.W. (2014). AMPK activation by isorhamnetin protects hepatocytes against oxidative stress and mitochondrial dysfunction. Eur. J. Pharmacol..

[145] Hu R., Wang M.Q., Liu L.Y., You H.Y., Wu X.H., Liu Y.Y., Wang Y.J., Lu L., Xiao W., Wei L.B. (2020). Calycosin inhibited autophagy and oxidative stress in chronic kidney disease skeletal muscle atrophy by regulating AMPK/SKP2/CARM1 signalling pathway. J. Cell. Mol. Med..

[146] Zeng C., Chen M. (2022). Progress in Nonalcoholic Fatty Liver Disease: SIRT Family Regulates Mitochondrial Biogenesis. Biomolecules.

[147] Dai H., Sinclair D.A., Ellis J.L., Steegborn C. (2018). Sirtuin activators and inhibitors: Promises, achievements, and challenges. Pharmacol. Ther..

[148] Tang X., Li X., Zhang D., Han W. (2022). Astragaloside-IV alleviates high glucose-induced ferroptosis in retinal pigment epithelial cells by disrupting the expression of miR-138-5p/Sirt1/Nrf2. Bioengineered.

[149] Yang C., Yang W., He Z., He H., Yang X., Lu Y., Li H. (2019). Kaempferol Improves Lung Ischemia-Reperfusion Injury via Antiinflammation and Antioxidative Stress Regulated by SIRT1/HMGB1/NF-κB Axis. Front. Pharmacol..

[150] Chang X., Zhang T., Meng Q., Wang S., Yan P., Wang X., Luo D., Zhou X., Ji R. (2021). Quercetin Improves Cardiomyocyte Vulnerability to Hypoxia by Regulating SIRT1/TMBIM6-Related Mitophagy and Endoplasmic Reticulum Stress. Oxidative Med. Cell. Longev..

[151] Wang J.Y., Nie Y.X., Dong B.Z., Cai Z.C., Zeng X.K., Du L., Zhu X., Yin X.X. (2021). Quercetin protects islet β-cells from oxidation-induced apoptosis via Sirt3 in T2DM. Iran. J. Basic Med. Sci..

[152] Oza M.J., Kulkarni Y.A. (2019). Formononetin attenuates kidney damage in type 2 diabetic rats. Life Sci..

[153] Zhai J., Tao L., Zhang S., Gao H., Zhang Y., Sun J., Song Y., Qu X. (2020). Calycosin ameliorates doxorubicin-induced cardiotoxicity by suppressing oxidative stress and inflammation via the sirtuin 1-NOD-like receptor protein 3 pathway. Phytother. Res..

[154] Zhang B., Hao Z., Zhou W., Zhang S., Sun M., Li H., Hou N., Jing C., Zhao M. (2021). Formononetin protects against ox-LDL-induced endothelial dysfunction by activating PPAR-γ signaling based on network pharmacology and experimental validation. Bioengineered.

[155] Ma Z., Ji W., Fu Q., Ma S. (2013). Formononetin inhibited the inflammation of LPS-induced acute lung injury in mice associated with induction of PPAR gamma expression. Inflammation.

[156] Liu Y., Chen L., Wu H., Zhang H. (2022). Delivery of astragalus polysaccharide by ultrasound microbubbles attenuate doxorubicin-induced cardiomyopathy in rodent animals. Bioengineered.

[157] Ou L., Wei P., Li M., Gao F. (2019). Inhibitory effect of Astragalus polysaccharide on osteoporosis in ovariectomized rats by regulating FoxO3a/Wnt signaling pathway. Acta Cir. Bras..

[158] Hu W., Zhang J., Wang H., Guan M., Dai L., Li J., Kang X. (2023). Protective effects of isorhamnetin against H_2_O_2_-induced oxidative damage in HaCaT cells and comprehensive analysis of key genes. Sci. Rep..

[159] Zhang M., Wang Y., Zhu G., Sun C., Wang J. (2021). Hepatoprotective effect and possible mechanism of phytoestrogen calycosin on carbon tetrachloride-induced liver fibrosis in mice. Naunyn-Schmiedeberg’s Arch. Pharmacol..

